# Terminal V5 Tagging of All Four Newcastle Disease Virus Structural Proteins Is Compatible with Particle Formation in Sf9 Cells

**DOI:** 10.3390/ijms27188181

**Published:** 2026-09-14

**Authors:** Aurelia Schweda, Dzmitry Dauhalevich, Lukasz Rabalski

**Affiliations:** 1 Laboratory of Recombinant Vaccines, Intercollegiate Faculty of Biotechnology, University of Gdansk and Medical University of Gdansk, Abrahama 58, 80-307 Gdansk, Poland; 2 Laboratory of Virus Molecular Biology, Intercollegiate Faculty of Biotechnology, University of Gdansk and Medical University of Gdansk, Abrahama 58, 80-307 Gdansk, Poland; 3 Biological Threats Identification and Countermeasure Center, General Karol Kaczkowski Military Institute of Hygiene and Epidemiology, Lubelska 4, 24-100 Pulawy, Poland; 4 VAXiCAN Sp. z o.o., Kampinoska 25, 80-180 Gdansk, Poland

**Keywords:** Newcastle disease virus, virus-like particles, V5 epitope tag, baculovirus expression vector system, Sf9 cells, particle assembly, transmission electron microscopy

## Abstract

Newcastle disease virus-like particles (NDVLPs) assemble from the matrix (M), nucleoprotein (NP), fusion (F) and hemagglutinin–neuraminidase (HN) proteins. Terminal epitope tags make these proteins easy to detect but may interfere with assembly, and it is not well-documented how much terminal modification the four proteins tolerate. Eight pFastBac Dual donor plasmids were used to generate recombinant baculoviruses, and twelve NDVLP variants were produced in Sf9 cells and purified by sucrose gradient ultracentrifugation. Particle-like structures were recovered from all twelve preparations. Western blotting confirmed production of the native and V5-tagged proteins and their co-purification in the particle fractions; transmission electron microscopy showed enveloped, roughly spherical particles with measured diameters of 55 to 150 nm (median 90 nm) in every preparation, similar in appearance to native NDV. Particles of a similar size were also present in the beta-glucuronidase control preparation, so the assignment of the particles rests on the protein composition of the fractions rather than on morphology alone. Particles formed regardless of which structural protein carried the tag, including a variant in which all four proteins were tagged simultaneously. Under the conditions tested, 19-residue terminal insertions were compatible with NDVLP recovery at each of the four positions examined.

## 1. Introduction

Newcastle disease virus (NDV) is an enveloped single-stranded negative-sense RNA virus assigned to the species *Orthoavulavirus javaense* (formerly *Avian orthoavulavirus 1*) under the current ICTV taxonomy, genus *Orthoavulavirus*, subfamily *Avulavirinae*, family *Paramyxoviridae* [1,2,3]. NDV infects a wide range of avian species, causing highly contagious Newcastle disease (ND), which leads to economic losses worldwide [4,5]. In contrast to the severe clinical signs observed in infected birds, transmission of NDV to humans is rare and typically results in mild, self-limiting symptoms [2,6,7,8].

Virus-like particles (VLPs) are non-infectious, self-assembling structures composed of one or more viral structural proteins that mimic the structure and size of a native virion particle. Due to their ability to present repetitive epitopes in their native conformation, VLPs can efficiently elicit high levels of humoral and cellular immune responses [9,10,11,12]. Compared to subunit vaccines, VLPs confer higher immunogenicity and antigenicity, while maintaining a superior safety profile relative to live-attenuated or inactivated viral vaccines [13,14].

NDV replication is restricted to avian hosts and productive infection of humans is rare, which supports the use of NDV-derived virus-like particles (NDVLPs) in vaccine research; inactivated NDV-vectored vaccines have been well-tolerated in human clinical studies [15]. Owing to their structural flexibility, NDVLPs can be engineered to incorporate foreign antigens on the particle surface [16,17,18,19,20].

Several studies have investigated NDVLP assembly. Efficient particle assembly has been consistently reported, although most studies used recombinant NDV or foreign proteins [10,21,22,23,24,25,26]. The requirements for the assembly of NDVLPs from the four structural proteins were defined by Pantua et al., who identified the M protein as necessary and sufficient for budding [27]; particles containing NP, M, HN and F released from avian cells were subsequently characterized in detail [23]. Most of these studies used avian or insect cell lines, with four generating NDV-derived VLPs in a baculovirus expression vector system (BEVS) [21,24,25,26]. Reported particles resembled authentic virions in density and release efficiency [27], and diameters of approximately 100 to 200 nm have been described [26]. It is, however, not documented how NDVLP assembly behaves when several structural proteins are modified at the same time. This study describes the construction of eight donor plasmids and the production of twelve NDVLP variants that differ in the number and position of terminal epitope tags. The aim was to survey the positions at which a short terminal epitope tag can be placed on the four structural proteins without preventing particle formation, rather than to characterize the resulting particles in depth.

## 2. Results

### 2.1. Confirmation of Native and Chimeric NDV Proteins Production

**Figure 1:**
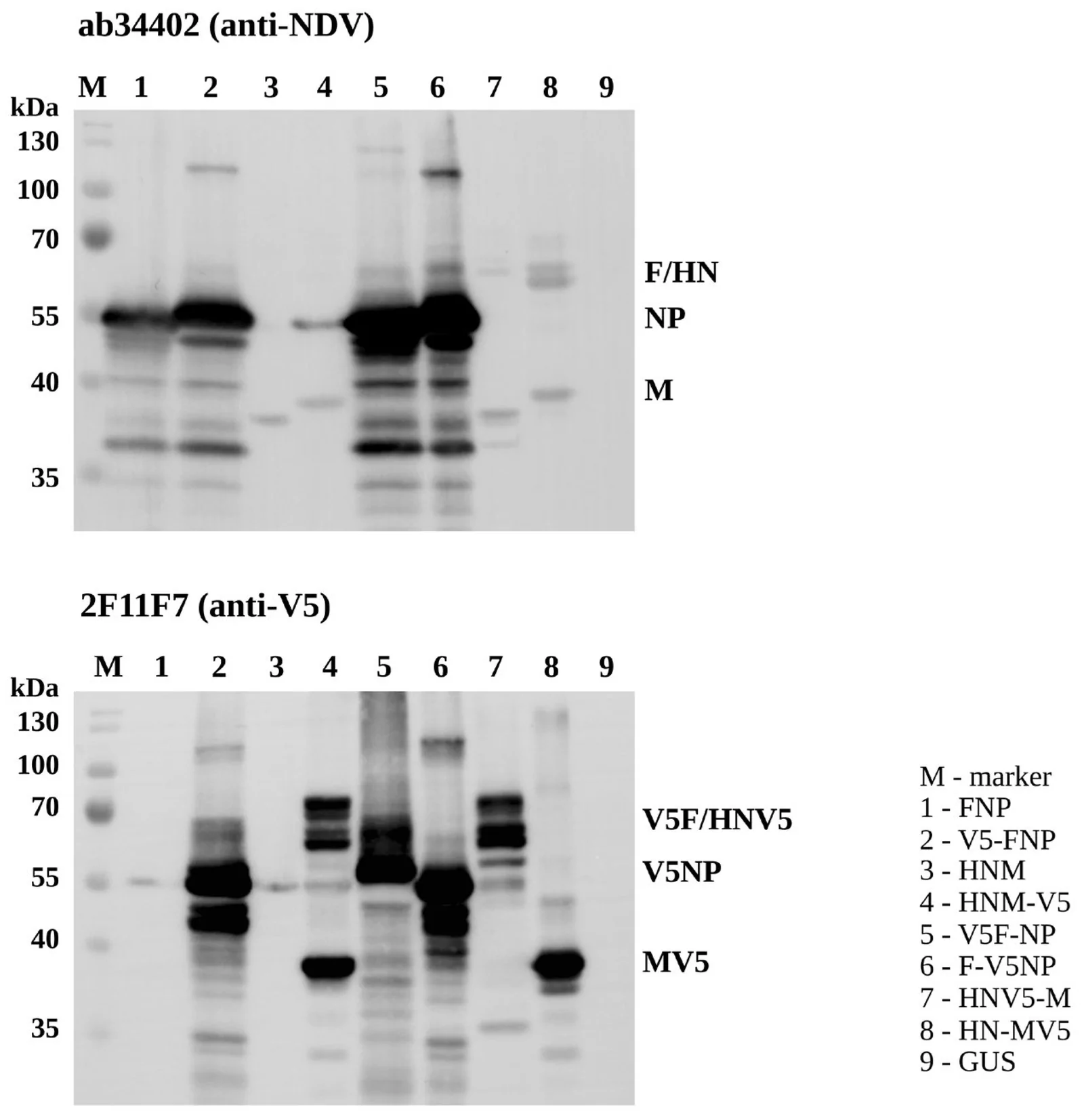
Western blot analysis of structural native and chimeric NDV proteins expression in Sf9 cells. Cellular fractions were harvested 5 days post-infection with P0 baculoviral stocks. Top panel: immunodetection of native proteins using a polyclonal anti-NDV (ab34402) antibody. Bottom panel: immunodetection of chimeric variants of NDV proteins using a monoclonal anti-V5 (2F11F7) antibody. Lanes: M: PageRuler Prestained Protein Ladder (Thermo Fisher Scientific, Waltham, MA, USA); 1: cells infected with baculovirus FNP; 2: V5-FNP; 3: HNM; 4: HNM-V5; 5: V5F-NP; 6: F-V5NP; 7: HNV5-M; 8: HN-MV5; 9: cells infected with baculovirus harboring the beta-glucuronidase gene (*GUS*) as a negative control. In the anti-V5 panel, lanes 2, 5 and 6 are saturated. A faint signal at the V5NP position is visible in lanes 1 and 3, which contain untagged proteins only; it is present in the original image and, given the saturation of the neighboring lanes, is most consistent with lateral spillover. Signals shown are from a single representative experiment; expression of each construct was confirmed in three to four independent experiments. Uncropped membrane images are provided in the Supplementary Materials (Figures S1 and S2).

Representative Western blot results are presented in Figure 1. Both unmodified and V5-tagged M, HN, F, and NP proteins were detected in cellular fractions using anti-NDV and anti-V5 antibodies, respectively. Because the P0 stocks used in this experiment were not titrated and equal volumes rather than equal protein amounts were loaded, band intensities are not compared between lanes. In contrast, native F in the FNP sample and native HN in the HNM sample were detected only weakly. This may reflect limited reactivity of the polyclonal ab34402 antibody with insect-cell-derived glycoproteins, lower expression levels, or both. The chimeric variants migrated at the positions expected for the tagged proteins; the 19-residue tag cassette adds approximately 2 kDa, which is close to the resolution limit of the gels used. The banding patterns of the chimeric proteins corresponded to those of their native counterparts, including the glycoform heterogeneity of the F and HN glycoproteins. In summary, our results confirm the successful production of native and chimeric variants of NDV M, HN, F, and NP proteins in the BEVS.

### 2.2. Characterization of Purified NDVLP Variants

To evaluate the ability of the proteins to self-assemble into NDVLPs and the impact of the modifications on particle structure, all purified protein samples were analyzed for both protein composition and morphological features.

#### 2.2.1. Analysis of Protein Composition

**Figure 2:**
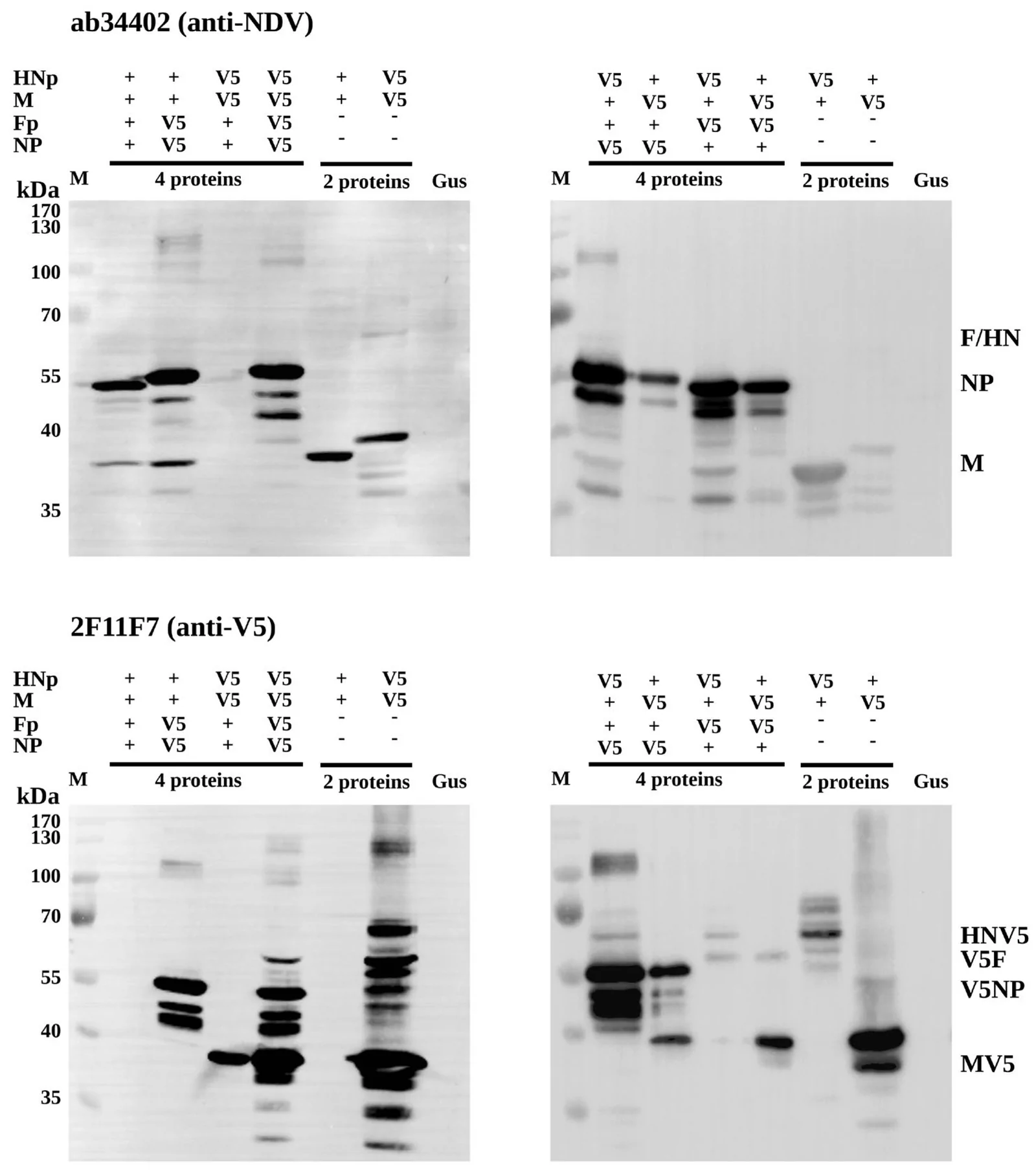
Western blot analysis of the protein composition of purified native and V5-tagged NDVLPs. Extracellular particles were purified from culture supernatants harvested 5 days post-infection with P2 or P3 rBV stocks, as specified in Section 4.4. Top panel: immunodetection of native NDV structural proteins using a polyclonal anti-NDV (ab34402) antibody. Bottom panel: immunodetection of chimeric proteins using a monoclonal anti-V5 (2F11F7) antibody. Individual lanes represent different NDVLP variants, with the protein composition indicated above each lane. M: PageRuler Prestained Protein Ladder. Purified culture supernatants from cells infected with an rBV expressing beta-glucuronidase (GUS) were used as a negative control. Left-hand panels show material recovered from the 50%/10% interface for six of the variants and right-hand panels show material recovered from the denser interface for the remaining six variants, which contained more residual cellular material. Each purified preparation was analyzed once, except for N-NDVLP and HNM VLP, which were analyzed twice. Each panel is a contiguous crop of a single membrane. Uncropped membrane images are provided in the Supplementary Materials (Figures S3–S6).

The protein composition of all purified samples was verified by Western blot analysis using anti-NDV and anti-V5 antibodies (Figure 2). The results confirmed the co-purification of the expected viral proteins in the generated NDVLP preparations, although the relative signal intensities varied between configurations.

Anti-V5 signals were detected in all preparations containing tagged proteins and were absent from preparations composed of native proteins only and from the GUS control. Loading was volume-based, the two antibodies were used at different dilutions and several anti-V5 lanes are saturated, so band intensities are not compared between lanes or between panels and no conclusion is drawn about the relative amount of protein incorporated into the particles (Section 4.5).

#### 2.2.2. Morphological Analysis of the Purified Preparations

**Figure 3:**
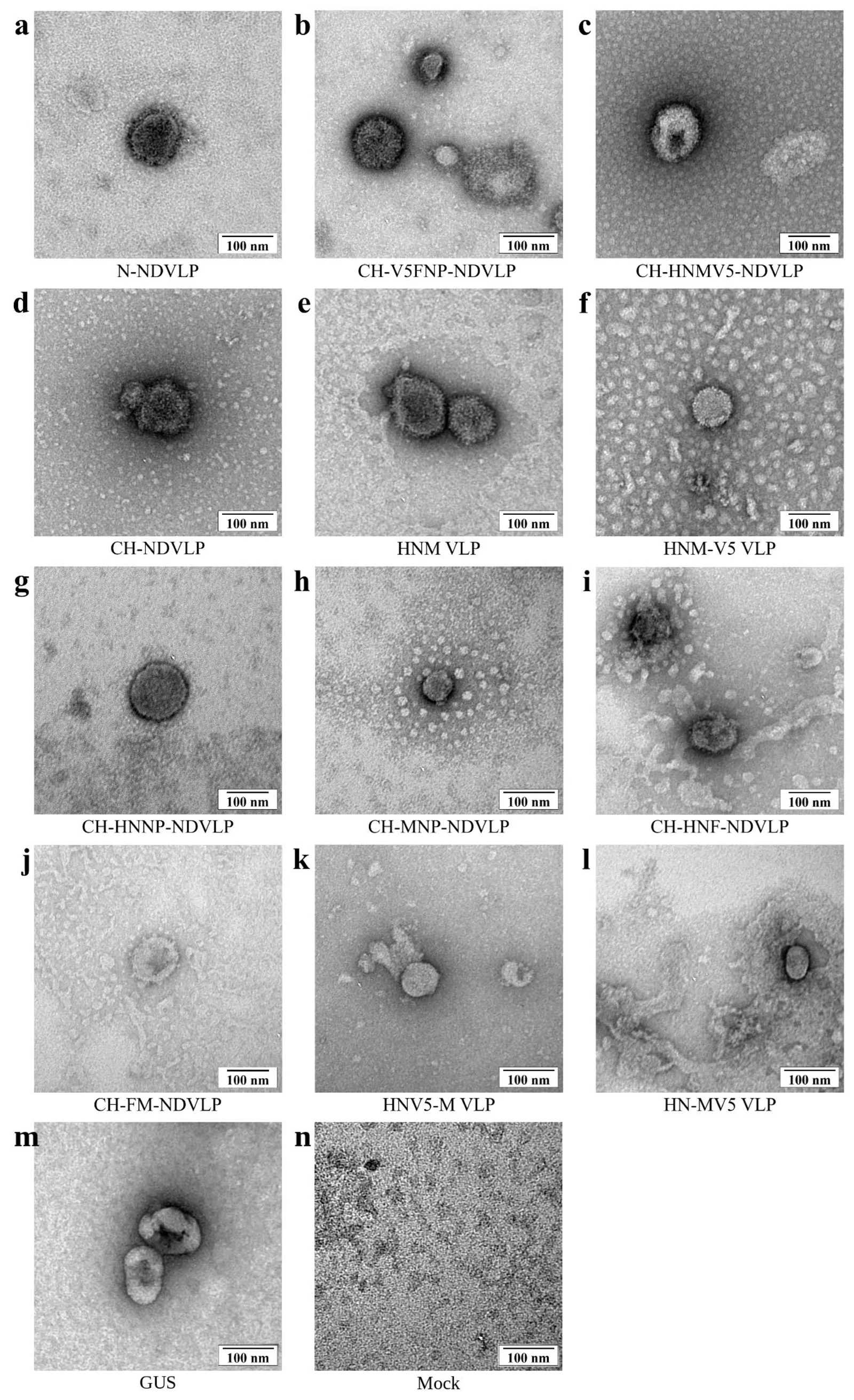
Characterization of purified native and V5-tagged NDVLPs by TEM. Panels (**a**–**l**) are labeled with the variant names defined in Table 1. Panels (**m**,**n**) show control preparations processed in parallel: (**m**) culture infected with the beta-glucuronidase-expressing baculovirus; (**n**) mock-infected culture. Scale bars = 100 nm. Micrographs of N-NDVLP, CH-V5FNP-NDVLP, CH-NDVLP, CH-MNP-NDVLP, HNM VLP, HN-MV5 VLP, HNV5-M VLP and both control preparations were recorded at 120,000× nominal magnification; micrographs of CH-HNMV5-NDVLP, CH-HNNP-NDVLP, CH-HNF-NDVLP, CH-FM-NDVLP and HNM-V5 VLP at 98,000×. One micrograph was recorded per preparation.

To confirm the formation of native and V5-tagged NDVLP variants and to characterize their morphology, purified samples were analyzed by transmission electron microscopy (TEM). Negatively stained preparations of all twelve samples contained enveloped, roughly spherical particles similar in appearance to native NDV particles (Figure 3a–l). Measured diameters ranged from 55 to 150 nm, with a median of 90 nm and an interquartile range of 80 to 105 nm. Particles of comparable appearance were observed in all preparations, irrespective of whether they were composed of native or V5-tagged proteins. Comparable size ranges have been reported for enveloped paramyxovirus-like particles produced in insect cells [28]. Particles 75 to 100 nm in diameter were also present in the GUS control preparation processed in parallel (Figure 3m), whereas none were observed in the mock-infected control (Figure 3n). The control particles differed in appearance from those in the NDVLP preparations: they showed folded, cup-shaped profiles with a stain-filled central depression, as is typical of extracellular vesicles collapsed during negative staining, whereas the particles in the NDVLP preparations more often had closed, convex outlines and granular, stain-excluding interiors.

**Table 1:** Native and chimeric NDVLP variants. The table details all twelve NDVLP variants, including their protein composition and the rBVs used for their production.

NDVLP Variant	Protein Components	V5 Tag Position	Recombinant Baculoviruses
N-NDVLP	HN, M, F, and NP	None	HNM and FNP
CH-HNMV5-NDVLP	HNV5, MV5, F, and NP	C-terminus (HN and M)	HNM-V5 and FNP
CH-V5FNP-NDVLP	HN, M, V5F, and V5NP	N-terminus (F and NP)	HNM and V5-FNP
CH-NDVLP	HNV5, MV5, V5F, and V5NP	C-terminus (HN and M) and N-terminus (F and NP)	HNM-V5 and V5-FNP
CH-MNP-NDVLP	HN, MV5, F, and V5NP	C-terminus (M) and N-terminus (NP)	HN-MV5 and F-V5NP
CH-FM-NDVLP	HN, MV5, V5F, and NP	C-terminus (M) and N-terminus (F)	HN-MV5 and V5F-NP
CH-HNNP-NDVLP	HNV5, M, F, and V5NP	C-terminus (HN) and N-terminus (NP)	HNV5-M and F-V5NP
CH-HNF-NDVLP	HNV5, M, V5F, and NP	C-terminus (HN) and N-terminus (F)	HNV5-M and V5F-NP
HNM VLP	HN and M	None	HNM
HNM-V5 VLP	HNV5 and MV5	C-terminus (HN and M)	HNM-V5
HN-MV5 VLP	HN and MV5	C-terminus (M)	HN-MV5
HNV5-M VLP	HNV5 and M	C-terminus (HN)	HNV5-M

## 3. Discussion

Virus-like particles are widely used as vaccine platforms, as they mimic the structural and immunogenic properties of the cognate infectious virus while eliminating the risks associated with viral replication. Among enveloped virus-like particles, NDVLPs have attracted increasing interest, and NDV-based vaccines have been well-tolerated in human clinical studies [15,29,30,31,32].

While the fundamental principles of NDV and NDV-derived VLP assembly have been studied [27,33], most work to date has used individual recombinant proteins or foreign antigens [16,21,22,24,25,26]. In the present study, all twelve variants, including the variant in which all four structural proteins carried the epitope tag, yielded particles of comparable appearance, with measured diameters of 55 to 150 nm (median 90 nm), as shown in Figure 3. These observations are consistent with the NDV structural proteins used here accommodating 19-residue terminal insertions, provided that the domains responsible for the protein–protein interactions required for particle assembly are retained.

Among recombinant expression systems, the BEVS is a well-established manufacturing platform. This insect cell-based system enables the large-scale production of recombinant proteins with complex post-translational modifications, which can be relevant for the antigenic properties of NDVLPs [34]. In our study, we utilized a dual promoter vector to co-express two proteins in the Sf9 insect cell line using the Bac-to-Bac baculovirus system, which is particularly effective for producing multicomponent particles. However, the native glycoproteins were detected only weakly in the FNP and HNM constructs. Unstable expression and reduced glycoprotein yields are recognized limitations of this system [35]. Contemporary BEVS processes nonetheless reach multi-milligram per liter yields of virus-like particles in insect cells [36], and enveloped particles assembled from several structural proteins delivered by separate recombinant baculoviruses have been produced in Sf9 cells at bioreactor scale [37].

Furthermore, the discrepancy in detection levels between native and chimeric forms highlights an important diagnostic limitation. Despite its well-documented advantages, the BEVS faces several challenges, including differences in glycosylation patterns between insect and mammalian cells [34]. Such post-translational modifications can impair antigenic presentation by reducing epitope accessibility, thereby preventing high-affinity binding of the polyclonal antibodies used. Incorporation of the V5 epitope at the termini of the recombinant proteins provided an alternative strategy for detecting them. A further option is the use of stably transformed insect cell lines with modified glycosylation, for example, *Drosophila melanogaster* S2+ cells in which the *fused lobes* (*FDL*) gene has been inactivated, which shifts the N-glycan profile away from paucimannosidic structures towards mammalian-type glycans [34].

Assembly of enveloped VLPs is more demanding than assembly of non-enveloped particles, because several membrane-anchored proteins must be brought together in a single lipid envelope [38]. The tag cassette used here is short, with 19 residues in total (a five-residue GPRFE linker and the 14-residue V5 epitope), and represents a relatively low steric burden, so the present results do not indicate how larger insertions would behave. Particle diameters were pooled across variants and the comparison between variants remains qualitative. Particles of a similar size range, though of different appearance, were recovered from control cultures infected with an irrelevant baculovirus, so the gradient fractions are not free of extracellular vesicles and the assignment of the particles rests on the recovery of the NDV structural proteins from a defined interface of an isopycnic gradient, after two consecutive gradients and a concentration step, rather than on morphology alone. No NDV-specific or V5-specific signal was detected in the control fraction, indicating that the presence of vesicles alone does not generate the observed signals. Whether the same configurations tolerate larger payloads, and how the tags affect immunogenicity, remains to be tested.

Three limitations bound these conclusions. First, none of the methods used assigns a given protein to an individual particle; orthogonal approaches such as nanoparticle tracking analysis, size-exclusion chromatography coupled to multi-angle light scattering, or immunogold labeling would be required to establish particle identity directly, and were outside the scope of this survey. Second, band intensities are not quantitative here, because loading was volume-based and the two antibodies were used at different dilutions. Third, each of the twelve variants was produced and purified once and one micrograph was recorded per preparation; the reproducibility of protein expression, but not of particle production, was assessed independently.

## 4. Materials and Methods

### 4.1. Insect Cell Lines

Two insect cell lines, *Spodoptera frugiperda* 9 (Sf9) and Sf9 Easy Titer Cell Line (Sf9-ET) [39], were used for NDVLPs production in a baculovirus expression system and viral titration, respectively. Both cell lines were cultured under standard conditions in HyQ SFX-Insect medium (Cytiva, Marlborough, MA, USA) in shake flasks at 27 °C with agitation at 130 rpm. The medium was supplemented with an Antibiotic-Antimycotic solution for the Sf9 insect cells and with the Geneticin Selective Antibiotic (Thermo Fisher Scientific, Waltham, MA, USA) for the Sf9-ET insect cells. Both cell lines are maintained in the laboratory collection of the Laboratory of Virus Molecular Biology and were obtained through scientific exchange rather than by commercial purchase; records of the original supplier are no longer available.

### 4.2. Recombinant pFastBac Dual Donor Plasmids

Coding sequences of the M, HN, F and NP proteins of NDV strain LaSota (GenBank accession no. AY845400.2; protein accession nos. AAW30678.1, AAW30680.1, AAW30679.1 and AAW30676.1, respectively) were codon-optimized for expression in *Spodoptera frugiperda* cells in Geneious version 9.0.5 and obtained by chemical gene synthesis (Thermo Fisher Scientific, Waltham, MA, USA). Throughout this work, native refers to the untagged forms of the proteins; all coding sequences used, including those carried by the plasmid FHNMNP, were codon-optimized synthetic genes. The F protein of this strain is of the lentogenic type, with the monobasic cleavage motif 112-GRQGR/L-117. Native gene fragments were amplified from the plasmid FHNMNP with Q5 High-Fidelity DNA Polymerase (New England Biolabs, Ipswich, MA, USA). Genes encoding the chimeric M-V5, HN-V5, V5-F and V5-NP proteins were generated by three successive PCR reactions performed on the single-gene plasmids pFastBac M, pFastBac HN, pFastBac F and pFastBac NP, using primer sets that introduced a five-amino-acid linker (GPRFE) and the V5 epitope (GKPIPNPLLGLDST) at the selected terminus. Both sets of plasmids had been generated previously in the laboratory. The M and HN proteins were tagged at the C-terminus, and F and NP at the N-terminus; in the V5-F and V5-NP constructs the initiation codon of the tag cassette replaces the native initiation codon, so that the NDV-derived part of the protein begins at residue 2. Reactions for the four chimeric constructs were performed separately, and the identity of all inserts was confirmed by Sanger sequencing (Eurofins Genomics, Ebersberg, Germany).

The amplicons were digested with EcoRI/HindIII (HN, HN-V5, F, V5-F) or KpnI/XhoI (M, M-V5, NP, V5-NP) and cloned into the pFastBac Dual Expression Vector (Gibco, Thermo Fisher Scientific, Waltham, MA, USA) digested with the corresponding restriction enzymes. Recombinant donor plasmids encoding two native NDV proteins (pFastBac-Dual-HNM and pFastBac-Dual-FNP) and two chimeric proteins (pFastBac-Dual-HNM-V5 and pFastBac-Dual-V5-FNP) were generated. The correctness of the assembly process was confirmed by restriction analysis.

**Figure 4:**
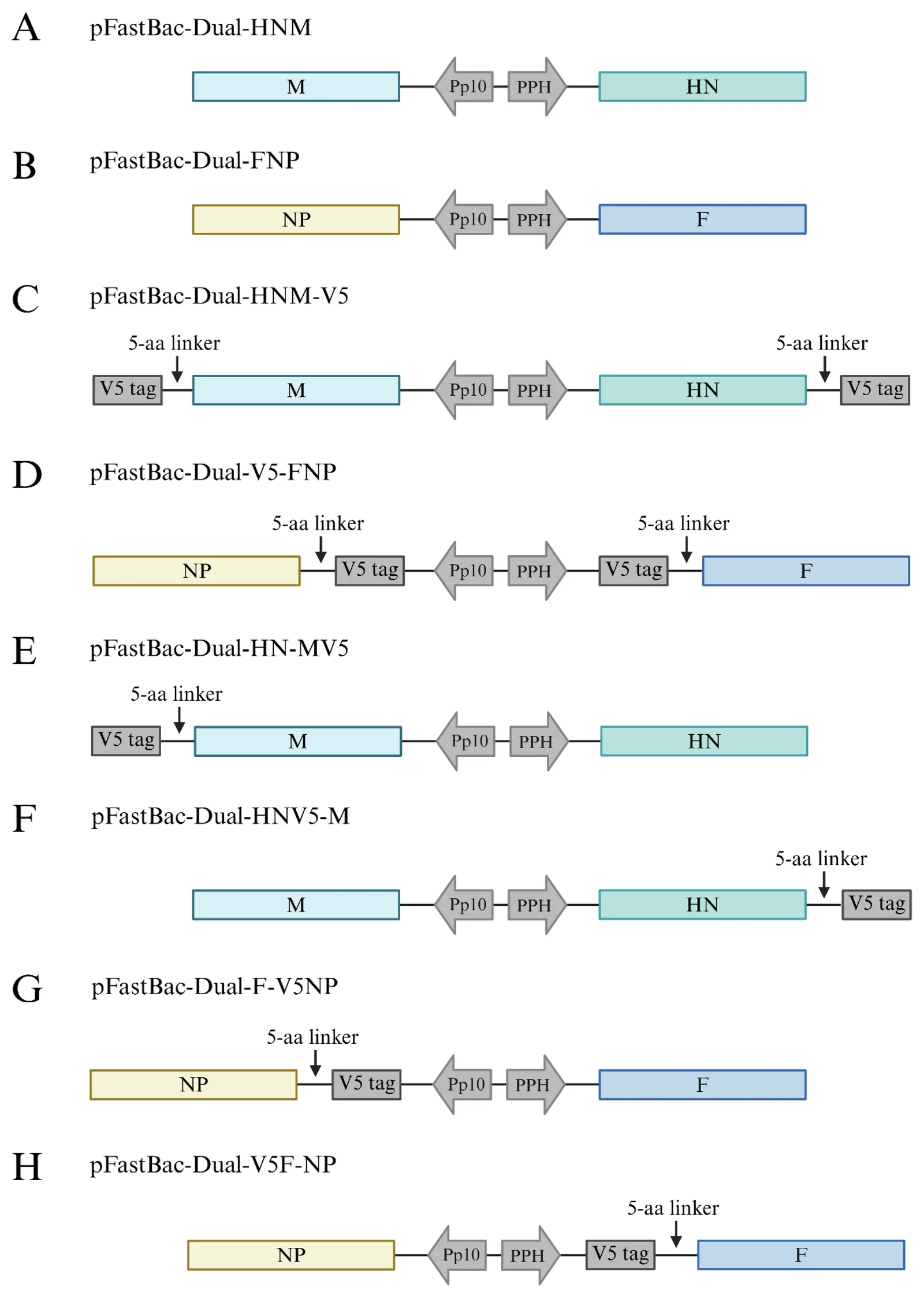
Schematic representation of the recombinant pFastBac Dual donor plasmids. For expression cassettes (**A**–**D**), genes encoding HN, F, HN-V5, and V5-F proteins were cloned downstream of the PH promoter (PPH) using EcoRI and HindIII restriction sites. The resulting plasmids were subsequently digested with KpnI/XhoI for insertion of M, NP, M-V5, and V5-NP coding sequences under the p10 promoter (Pp10), respectively. Constructs (**E**–**H**) were generated by subcloning using the appropriate restriction enzymes. Created in BioRender. Schweda, A. (2026) https://BioRender.com/fhyuk9v.

Subsequently, subcloning was performed and an additional four donor plasmids encoding combinations of native and chimeric forms of the proteins were obtained (pFastBac-Dual-HN-MV5, pFastBac-Dual-HNV5-M, pFastBac-Dual-F-V5NP, and pFastBac-Dual-V5F-NP). A schematic representation of all constructed donor plasmids is shown in Figure 4.

### 4.3. Recombinant Bacmids and Baculoviruses

Recombinant pFastBac Dual donor plasmids containing full-length wild-type NDV *M* and *HN* or *NP* and *F* genes, V5-tagged chimeric proteins, or combinations of native and chimeric forms were used for site-specific transposition into the MAX Efficiency DH10Bac *E. coli* strain using the Bac-to-Bac Baculovirus Expression System according to the manufacturer’s instructions (Thermo Fisher Scientific, Waltham, MA, USA); the system is based on the method described by Luckow et al. [40]. Bacmid DNA was isolated using the ZR BAC DNA Miniprep Kit (Zymo Research, Irvine, CA, USA), and the presence of successful insertion of expression cassettes was confirmed by PCR using the universal M13 forward and reverse primers. The amplification products were analyzed by agarose gel electrophoresis.

Recombinant P0 baculovirus stocks were generated by transfecting Sf9 cells with all eight recombinant bacmid DNA constructs (HNM, FNP, HNM-V5, V5-FNP, HN-MV5, HNV5-M, F-V5NP, and V5F-NP) using the Transporter 5 Transfection Reagent (Polysciences, Warrington, PA, USA). Culture supernatants were harvested 5 days post-transfection, tested for the presence of rBVs by Dot blot analysis using an anti-gp64 antibody (Invitrogen, Waltham, MA, USA), and subsequently amplified by three passages in Sf9 cells to achieve higher titers. The functional titers of P2 and P3 viral stocks were determined using Sf9-ET cells as described previously [39].

### 4.4. Production and Purification of Native and Chimeric NDVLPs

Sf9 cells, grown in suspension culture at a density of 2 × 10^6^ cells/mL in a 30 mL working volume, were either infected individually or co-infected with the recombinant baculoviruses at a multiplicity of infection (MOI) of 2.5 per construct, corresponding to a total MOI of 5 in co-infections. The specific rBVs used for the production of each NDVLP variant are presented in Table 1. Production was carried out with P3 rBV stocks, except for CH-MNP-NDVLP and HN-MV5 VLP, for which P2 stocks were used, and CH-FM-NDVLP and CH-HNNP-NDVLP, for which one P2 and one P3 stock were combined (HN-MV5 at P2 with V5F-NP at P3, and F-V5NP at P2 with HNV5-M at P3). In each case, the passage with the higher functional titre was selected. Five days post-infection, the viral cultures were harvested and centrifuged at 5000 rpm for 15 min at 4 °C to remove cells and cellular debris.

Clarified cell culture supernatants were pelleted by ultracentrifugation at 14,600 rpm (approximately 38,000× *g*) overnight at 4 °C in an SW28 swinging-bucket rotor (Beckman Coulter, Brea, CA, USA). The pellets were resuspended in TNE buffer (150 mM NaCl, 25 mM Tris-HCl pH 7.4 and 5 mM EDTA) and layered onto discontinuous sucrose gradients composed of 2 mL of 65% and 4 mL of 20% sucrose (*w*/*v*). The gradients were ultracentrifuged in an SW41 Ti swinging-bucket rotor (Beckman Coulter) at 25,400 rpm (approximately 110,000× *g*) for 6 h at 4 °C. The white layers between the 65% and 20% sucrose layers were collected, mixed with 3 mL of 80% sucrose, layered beneath a final isopycnic gradient consisting of 0.5 mL of 80% sucrose, and then sequentially overlaid with sucrose layers of 3.5 mL of 50% and 2 mL of 10% sucrose (*w*/*v*). The gradients were ultracentrifuged in an SW41 Ti rotor at 35,000 rpm (approximately 210,000× *g*) overnight at 4 °C. Two white layers formed in this gradient and both were collected and analyzed separately. The sucrose fractions were diluted with 8 mL of TNE buffer and concentrated by final ultracentrifugation in the SW41 Ti rotor at 34,000 rpm (approximately 198,000× *g*) for 6 h at 4 °C. The pellets were resuspended in 100 μL of 10% sucrose solution and tested for the presence of NDV native and chimeric proteins by sodium dodecyl sulfate polyacrylamide gel electrophoresis (SDS-PAGE) followed by Western blot analysis.

Mock-infected cells and cultures infected with a baculovirus expressing the beta-glucuronidase enzyme (GUS) were used as controls and were prepared in the same way as the analyzed samples. The GUS control was included in the Western blot analyses; both controls were examined by transmission electron microscopy in parallel with the samples.

### 4.5. Western Blot Analysis of Heterologous Protein Production and VLP Composition

Western blot analysis was performed to confirm both the expression of unmodified and V5-tagged structural NDV proteins in Sf9 insect cells, and to verify the protein composition of the final purified protein samples. Cellular fractions (harvested 5 days post-infection with P0 baculoviral stocks) and purified NDVLP samples were separated on 12% acrylamide gel under denaturing conditions and transferred to a polyvinylidene fluoride (PVDF) membrane. For the analysis of protein production, cell pellets from 5 mL of culture (5 × 10^6^ cells infected with 300 μL of P0 stock) were harvested 5 days post-infection and resuspended in 500 μL of 1× TNE buffer, and 2 μL of the suspension was loaded per lane. For the analysis of particle composition, 4 μL of the purified material resuspended in 10% sucrose was loaded per lane. Protein concentrations of the twelve purified preparations, estimated by absorbance at 280 nm (DeNovix spectrophotometer; DeNovix Inc., Wilmington, DE, USA), ranged from 2.9 to 19.5 mg/mL. Band intensities do not report the relative abundance of the proteins between lanes. Western blot analysis was performed using two commercially available antibodies, anti-NDV polyclonal antibody (cat no. ab34402, Abcam, Cambridge, UK) and anti-V5 Tag Monoclonal Antibody (2F11F7, cat no. 37-7500, Invitrogen) for detection of native and chimeric NDV proteins, respectively. The antibodies were diluted in blocking buffer at a ratio of 1:250 for ab34402 and 1:750 for 2F11F7. The protein bands were visualized using the Alliance Q9 imaging system (Uvitec, Cambridge, UK) after reaction with the SuperSignal West Pico PLUS Chemiluminescence Substrate (cat no. 34580, Thermo Fisher Scientific). Expression of the eight rBV-encoded protein pairs was assessed in three to four independent experiments per construct, each starting from an independent baculovirus generation and infection. Each of the twelve NDVLP variants was produced and purified once; purified preparations were analyzed by Western blotting once, except for N-NDVLP and HNM VLP, which were analyzed twice. Control preparations were produced twice. Figure 1 and Figure 2 show cropped regions of the membranes; all panels are contiguous crops of single membranes and no lanes were removed from within the displayed regions. Uncropped membrane images corresponding to Figure 1 are provided in Figures S1 and S2, and those corresponding to Figure 2 in Figures S3–S6 (Supplementary Materials).

### 4.6. Transmission Electron Microscopy of Purified VLPs

The self-assembly of all twelve NDVLPs and their morphology were analyzed using a transmission electron microscope (TEM). Purified native and chimeric NDVLPs suspended in 10% sucrose solution were diluted 30-fold in ultrapure water and applied to nickel grids coated with carbon film (Electron Microscopy Sciences, Hatfield, PA, USA). The grids were subsequently contrasted for 1 min with a 2% aqueous solution of uranyl acetate. Images were captured using a Tecnai G2 Spirit BioTWIN transmission electron microscope (FEI, Hillsboro, OR, USA) operated at 120 kV, at nominal magnifications of 98,000× and 120,000×. Particle diameters were measured on the original micrographs. Each micrograph was calibrated against its own scale bar, which corresponded to 212 pixels at 120,000× and 173 pixels at 98,000×; the ratio of the two bar lengths agreed with the ratio of the nominal magnifications to within 0.1%. Diameters were read to the nearest 10 nm as the outer boundary of the particle against calibrated concentric overlay circles spaced 20 nm apart. Particles cut by the image edge were excluded, and lobes of contacting clusters were measured individually only where their outlines could be traced.

## 5. Conclusions

In this study, eight pFastBac Dual donor plasmids and the corresponding recombinant baculoviruses were used to produce twelve NDVLP variants in Sf9 cells. All twelve preparations contained enveloped particles of comparable appearance, which, taken together with the co-purification of the corresponding structural proteins, are consistent with 19-residue terminal epitope insertions in the M, HN, F and NP proteins being compatible with particle formation under the conditions tested, both in two-protein particles carrying a single tagged protein and in four-protein particles in which up to all four proteins are tagged. The tested insertions were 19 amino acids long, and the tolerance of the system to larger modifications was not addressed. These observations are relevant to the production of VLP-based antigens in insect cell systems.

## Data Availability

The original contributions presented in this study are included in the article/Supplementary Materials. Further inquiries can be directed to the corresponding author.

## Supplementary Materials

The following supporting information can be downloaded at https://www.mdpi.com/article/10.3390/ijms27188181/s1.
