# Supplementary material for: Terminal V5 Tagging of All Four Newcastle Disease Virus Structural Proteins Is Compatible with Particle Formation in Sf9 Cells
Source: Int J Mol Sci. 2026 Sep 14;27(18):8181. doi: 10.3390/ijms27188181 (PMC13607146; doi:10.3390/ijms27188181)
Supplement: Supplementary file 1 [file ijms-27-08181-s001.zip › Supplementary_Materials_R1.pdf]

Supplementary Materials

Newcastle disease virus structural proteins: uncropped Western blot membranes

Manuscript ijms-4501549. The files listed below are the original, unmodified images acquired on the Alliance Q9 imaging system. Figures 1 and 2 of the manuscript show cropped regions of these membranes. The membranes also carry lanes with samples that are not part of this study; these lanes are not shown in the manuscript figures and are not described here. The file names follow the figure numbering of the originally submitted version of the manuscript.

Figure S1. Figure 2_ab34402.Tif

Uncropped membrane corresponding to the upper panel of Figure 1 (polyclonal anti-NDV antibody, ab34402).

Lanes shown in Figure 1, from left: M, PageRuler Prestained Protein Ladder; 1, cells infected with baculovirus FNP; 2, V5-FNP; 3, HNM; 4, HNM-V5; 5, V5F-NP; 6, F-V5NP; 7, HNV5-M; 8, HN-MV5; 9, beta-glucuronidase (GUS) control. The remaining lanes of this membrane are not part of this study.

Figure S2. Figure 2_2F11F7.Tif

Uncropped membrane corresponding to the lower panel of Figure 1 (monoclonal anti-V5 antibody, 2F11F7).

Lane order as for Figure S1. Lanes 2, 5 and 6 are saturated. The remaining lanes of this membrane are not part of this study.

Figure S3. Figure 3_ab34402_VLPs (left).Tif

Uncropped membrane corresponding to the upper left panel of Figure 2 (anti-NDV, ab34402); material recovered from the 50%/10% sucrose interface.

Lanes shown in Figure 2, from left: M, ladder; 1, N-NDVLP; 2, CH-V5FNP-NDVLP; 3, CH-HNMV5-NDVLP; 4, CH-NDVLP; 5, HNM VLP; 6, HNM-V5 VLP; 7, beta-glucuronidase (GUS) control. The remaining lanes of this membrane are not part of this study.

Figure S4. Figure 3_2F11F7_VLPs (left).Tif

Uncropped membrane corresponding to the lower left panel of Figure 2 (anti-V5, 2F11F7); same fraction and same lane order as Figure S3.

The remaining lanes of this membrane are not part of this study.

Figure S5. Figure 3_ab34402_VLPs (right).Tif

Uncropped membrane corresponding to the upper right panel of Figure 2 (anti-NDV, ab34402); material recovered from the denser sucrose interface, second set of variants.

Lanes shown in Figure 2, from left: M, ladder; 1, CH-HNNP-NDVLP; 2, CH-MNP-NDVLP; 3, CH-HNF-NDVLP; 4, CH-FM-NDVLP; 5, HNV5-M VLP; 6, HN-MV5 VLP; and the beta-glucuronidase (GUS) control lane. The remaining lanes of this membrane are not part of this study.

Figure S6. Figure 3_2F11F7_VLPs (right).Tif

Uncropped membrane corresponding to the lower right panel of Figure 2 (anti-V5, 2F11F7); same fraction and same lane order as Figure S5.

The remaining lanes of this membrane are not part of this study. The GUS control gave no signal with either antibody on any of the six membranes.
